# Molecular pathogenesis and prognostication of "low‐grade'' and "high‐grade" endometrial stromal sarcoma

**DOI:** 10.1002/gcc.22907

**Published:** 2020-11-10

**Authors:** Francesca Micci, Sverre Heim, Ioannis Panagopoulos

**Affiliations:** ^1^ Section for Cancer Cytogenetics Institute for Cancer Genetics and Informatics Oslo Norway; ^2^ Institute of Clinical Medicine, Faculty of Medicine University of Oslo Oslo Norway

**Keywords:** chromosomal aberrations, endometrial stromal sarcoma, fusion gene, high‐grade ESS, low‐grade ESS

## Abstract

Endometrial stromal sarcomas (ESS) are a heterogeneous group of rare mesenchymal cancers. Considerable knowledge has been gained in recent years about the molecular characteristics of these cancers, which helps to classify them in a more meaningful manner leading to improved diagnosis, prognostication, and treatment. According to this classification, ESS is now grouped as low‐ or high‐grade. ESS may have overlapping clinical presentation, morphology, and immunohistochemical profile. Their genetic characteristics allow subdivision of many of them depending on which pathogenetically important fusion genes they carry, but clearly much more needs to be unraveled in this regard. We here provide an overview of the molecular pathogenetic knowledge gained so far on low‐ and high‐grade ESS.

## 1 INTRODUCTION

According to the latest World Health Organization (WHO) classification of tumors, Endometrial Stromal Sarcomas (ESS) belongs to the overall category of Endometrial Stromal and related Tumors (EST). The spectrum runs from the completely benign, that is endometrial stromal nodules (ESN) showing well‐circumscribed margins with cells resembling those of proliferative‐phase endometrial stroma, to malignant, high‐grade ESS (HG‐ESS), which show destructive growth with invasion of surrounding myometrium, to the highly aggressive, undifferentiated uterine sarcomas (UUS) showing high‐grade cytological features, but no specific type of differentiation. Thus, ESS are malignant tumors composed of cells resembling stromal cells of proliferative‐phase endometrium, with a tendency toward infiltrative growth into the myometrium and/or lymphovascular spaces. The HG variants show round cell morphology that may be associated with a low‐grade spindle cell component, which is frequently fibromyxoid.^1^ Though most of these tumors originate from the uterus, a subset arises in extrauterine locations, such as, the ovary or peritoneum, often in association with endometriosis.^2^, ^3^

ESS are rare, accounting for 7% to 25% of all uterine mesenchymal tumors or 1% of all malignancies arising in the uterus. They are the second most common uterine malignant mesenchymal tumors after leiomyosarcoma.^1^, ^2^ The morphologic features, clinical behavior, and genetic aberration pattern identified in ESS allowed for separation into two categories: high and low grade. However, the complexity and heterogeneity of these tumors extend far beyond this diagnostic grouping.

## 2 CHROMOSOMAL ABERRATIONS AND THEIR MOLECULAR PRODUCTS

Different types of chromosomal aberrations have been described in ESS, with the most common being translocations involving two different chromosomes. Regardless of whether the translocation is balanced or unbalanced, the molecular product of such rearrangements is usually a so‐called fusion gene. This is a hybrid formed from two previously independent genes. It has been known for more than 30 years that gene fusions play an important role in tumorigenesis.^4^, ^5^ Oncogenic fusions may lead to an abnormal gene product brought about by fusing elements from the two fusion partners. Alternatively, a proto‐oncogene may be fused to a strong promoter leading to its upregulation. Oncogenic fusion transcripts may also be caused by trans‐splicing or read‐through events. Identification of an activated fusion gene improves diagnostic precision as well as prognostication, while at the same time providing pathogenetic information about the tumor.^6^

Though different fusion genes have been identified in ESS, generally it seems that the presence of one fusion gene excludes the presence of another in the same tumor.

### 2.1 LG‐ESS

**FIGURE 1:**
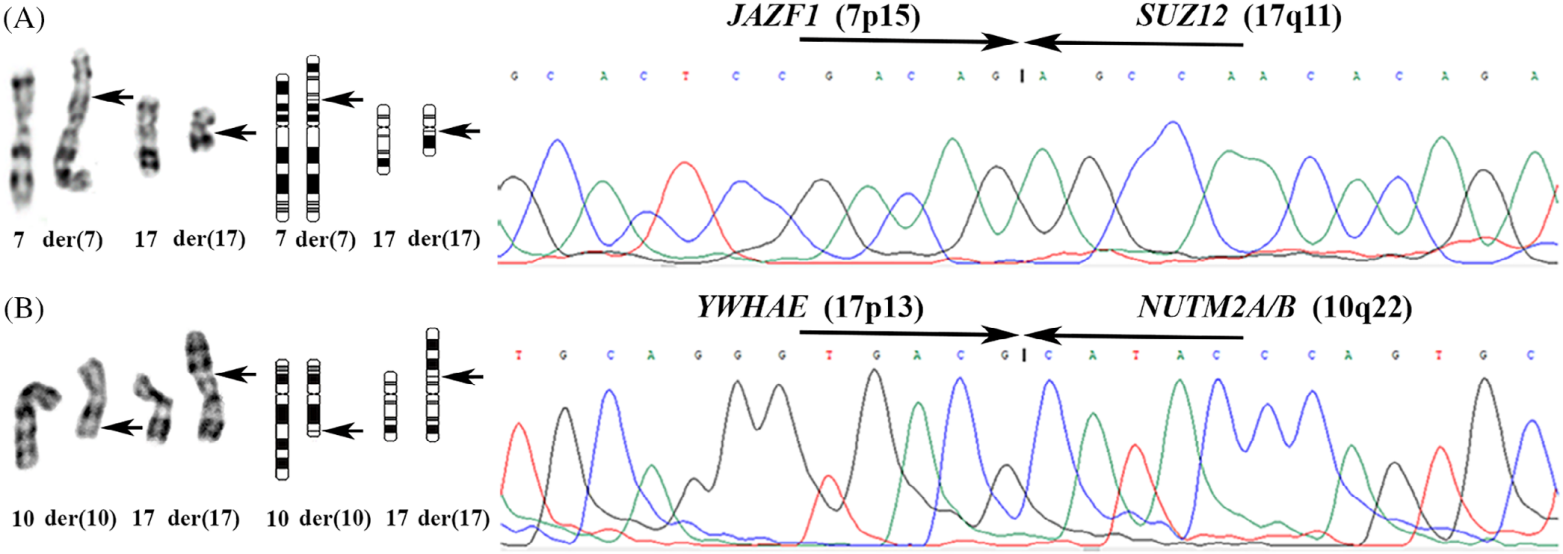
Partial karyogram and chromatogram of the hallmarks for ESS. A, LG‐ESS: partial karyogram showing the t(7;17)(p15;q11) (left), the ideograms for the rearranged chomosomes (center), and sequence chromatogram for the *JAZF1/SUZ12* fusion gene (right). B, HG‐ESS: partial karyogram showing the t(10;17)(q22;p13) (left), the ideograms for the rearranged chomosomes (center), and the sequence chromatogram for the *YWHAE/NUTM2A/B* fusion gene (right). Arrows point at breakpoints

Since 1988, when the first cytogenetic abnormalities were reported in ESS,^7^ many characteristic chromosomal rearrangements have been described in this group of tumors. The most distinctive cytogenetic hallmark of LG‐ESS is the 7;17‐translocation (Figure 1), first described by Sreekantaiah et al in 1991.^8^ The aberration leads at the molecular level to fusion of two zinc finger genes, *JAZF1* (from 7p15) and *SUZ12* (previously known as *JJAZ1*; from 17q21; Figure 1).^9^ Many other chromosomal changes have also been described and their pattern of occurrence is clearly nonrandom (Table 1). The molecular product behind each translocation has been identified for most of them. Ever improving methodological tools have facilitated the discoveries, especially the introduction of deep sequencing technologies allowing rapid screening of tumor genomes and transcriptomes.^10^ The second most common rearrangement involves chromosome band 6p21 and the *PHF1* gene.^11^ *PHF1* may recombine with several partners, not least *JAZF1* trough an unbalanced 6;7‐translocation.^11^ Other partners are *EPC1* through a 6;10‐rearrangement^11^; *MEAF6* through a t(1;6)(p34;p21)^12^; *BRD8* via t(5;6)(q31;p21)^13^; *EPC2* through a 2;6‐rearrangement^14^; and recently a *MBTD1/PHF1* was also reported.^15^ A study by D'Angelo et al.^16^ showed that tumors bearing *PHF1* fusions, independently of which partner gene is involved, typically present sex cord‐like differentiation, leading the authors to suggest that rearrangements of this gene preferentially induce such differentiation.

**TABLE 1:** Overview of chromosomal rearrangements and their respective fusion genes detected in low‐grade and high‐grade endometrial stromal sarcomas, and their occurrence in other types of neoplasms

ESS type	Chromosomal rearrangement	Fusion transcript and/or molecular aberration	Other neoplasm^a^
Low‐grade	t(7;17)(p15;q11)	*JAZF1/SUZ12*	ESN
Low‐grade	t(6;7)(p21;p15)	*JAZF1/PHF1*	cardiac ossifying sarcoma
Low‐grade	t(6;10)(p21;p11)	*EPC1/PHF1*	OFM
Low‐grade	t(1;6)(p34;p21)	*MEAF6/PHF1*	ESN, OFM
Low‐grade	t(X;17)(p11; q21)	*MBTD1/EZHIP*	
Low‐grade	t(5;6)(q13;p21)	*BRD8/PHF1*	
Low‐grade	ins(6;2)(p21;q23q23)	*EPC2/PHF1*	
Low‐grade	t(6;17)(p21;q21) putative^b^	*MBTD1/PHF1*	
Low‐grade	t(X;7)(q26;p15) putative	*JAZF1/BCORL1*	adenosarcoma
Low‐grade	t(1;17)(p34;q11) putative	*MEAF6/SUZ12*	
Low‐grade	t(10;17)(p11;q11) putative	*EPC1/SUZ12*	
Low‐grade	t(X;10)(p11;p11) putative	*EPC1/BCOR*	
High‐grade	t(10;17)(q22;p13)	*YWHAE/NUTM2A/B*	LMS, angiosarcoma, CCSK, SRBCS, URCS, PMMTI, NRCS,
High‐grade	t(X;22)(p11; q13)	*ZC3H7B/BCOR*	OFM
High‐grade	t(X;3)(p11;q28) putative	*LPP/BCOR*	
High‐grade		*BCOR* ITD	CCSK, PMMTI, RCS

^a^ CCSK, clear cell sarcoma of the kidney; ESN, endometrial stromal nodule; OFM, ossifying fibromyxoid tumor; LMS, leiomyosarcoma; NRCS, neonatal round cell sarcoma; PMMTI, primitive myxoid mesenchymal tumor of infancy; RCS, round cell sarcoma; SRBCS, small round blue cell sarcoma; URCS, undifferentiated round cell sarcoma.

^b^ Putative, the fusion was found by sequencing analysis and the chromosomal rearrangement designed by default.

A less frequent chromosomal rearrangement is the t(X;17)(p11;q21) leading to the *MBTD1/EZHIP* (previously known as *CXorf67*) fusion.^17^ Variants of the *JAZF1/SUZ12* were recently identified in which *JAZF1* recombines with *BCORL1* ^18^ and *SUZ12* with *MEAF6*.^19^

Another two novel chimeric fusions were reported by Dickson et al.,^20^ *EPC1/SUZ12* and *EPC1/BCOR*. The identification of these transcripts underlines the promiscuous nature of *EPC1*, but also obfuscates the molecular distinction between high grade and low grade ESS. Both tumors were described as clinically aggressive and with morphological features compatible with HG‐ESS.^20^ The biological potential associated with these fusions remains to be fully characterized. Most likely, also other fusion gene products will emerge. Until a sufficient number of cases is studied and the clinical parameters correlated, it is likely to remain challenging to classify the observed molecular events as being fully specific for LG‐ or HG‐ESS.

The above‐mentioned fusion genes have so far not been seen in leiomyomas, leiomyosarcomas, and uterine tumor resembling ovarian sex cord stromal tumors (UTROSCT), all of which may on occasion be differential diagnoses. Nevertheless, none of the fusions is fully pathognomonic for LG‐ESS as they have all been found also in other neoplasias. *JAZF1/SUZ12* fusion is detected in 65% to 75% of ESN.^9^, ^21^, ^22^, ^23^, ^24^, ^25^, ^26^, ^27^ This chimera has been found more frequently in classic LG‐ESS than in LG‐ESS exhibiting variant features.^28^ Recently, an ESN with *MEAF6/PHF1* was reported, providing further support for a continuum between these two tumor entities.^29^ Interestingly, the ESN showed focal peripheral ossification, a rare feature of ESN and/or LG‐ESS but a hallmark of ossifying fibromyxoid tumors with which they may share also other molecular events such as *PHF1* fusions, that is, *EP400/PHF1*, *MEAF6/PHF1*, and *EPC1/PHF1*. It was therefore hypothesized that the *MEAF6/PHF1* could be associated with metaplastic bone formation.^30^ The aforementioned fusions occur in ossifying fibromyxoid tumors of soft parts, irrespective of whether the tumor is diagnosed as typical, atypical, or malignant, whereas *JAZF1/PHF1* has been found in cardiac ossifying sarcomas.^31^, ^32^, ^33^, ^34^ Furthermore, two novel fusions, *CREBBP/BCORL1* and *KDM2A/WWTR1* have been reported in ossifying fibromyxoid tumors^35^ showing additional overlap with LG‐ESS as the genes *CREBBP* and *KDM2B* were previously found in a chimera in the latter tumor as well.^36^ Recently, the *JAZF1/BCORL1* fusion was identified in an adenosarcoma arising in the uterus.^37^

### 2.2 HG‐ESS

The cytogenetic hallmark of HG‐ESS is the balanced t(10;17)(q22;p13) translocation simultaneously reported in 2003 by two groups^38^, ^39^ (Figure 1). The gene product it leads to was identified by Lee et al.^40^ as an in‐frame fusion between the *YWHAE* and *NUTM2A/B* genes (previously known as *FAM22A/B*; Figure 1). The fusion seems to be specific for HG‐ESS as it was never identified in other gynecological tumors or neoplastic lesions, such as, uterine adenosarcomas, carcinosarcomas, leiomyosarcomas, leiomyomas, and polypoid endometriosis.^40^ Kubo et al.^41^ reported a low frequency of *YWHAE* and *NUTM2A/B* rearrangements in epithelioid leiomyosarcoma; admittedly, the immunostaining data of that study were suggestive of an unusual ESS. Splitting of probes for the *YWHAE*, *FAM22A*, and *FAM22B* genes has been reported in a uterine angiosarcoma.^42^ Despite the fact that no fusion transcript involving the mentioned genes was discovered, the authors suggested that abnormalities of them may contribute to development of uterine angiosarcoma in much the same manner as they do in ESS.^42^ Of further note in the context is the fact that no such rearrangement was identified in 21 angiosarcomas of extrauterine soft tissue.^40^ However, the very same chromosomal translocation has been reported in clear cell sarcomas of the kidney by different groups^43^, ^44^, ^45^ and shown to correspond to a *YWHAE/NUTM2A/B* fusion.^46^ Kao et al.^47^ identified it also in small round blue cell sarcomas of soft tissue, undifferentiated round cell sarcoma, and primitive myxoid mesenchymal tumor of infancy.^47^, ^48^ Recently, the first neonatal case of a round cell sarcoma bearing this chimera was described in a tumor with aggressive clinical behavior.^49^ Sciallis et al.^50^ studied 17 HG‐ESS and outlined three morphological patterns in this tumor type: *YWHAE* rearrangements were identified only in tumors showing high‐grade round cells with brisk mitotic activity and necrosis, not in all examined tumors.

A subset of tumors within the HG‐ESS category has lately been described as having aggressive behavior and mimicking myxoid leiomyosarcoma morphologically. These tumors show the *ZC3H7B/BCOR* fusion first reported by Panagopoulos et al.^51^ in two ESS showing a (X;22)(p11;q13) chromosomal translocation, a rearrangement later confirmed in several tumors by other investigators.^52^, ^53^, ^54^, ^55^ Most of the clinical data published on patients diagnosed with HG‐ESS showed stage 3 disease or the patient had a recurrence. However, ESS harboring a *ZC3H7B/BCOR* fusion may be clinically as well as morphologically heterogeneous.^54^, ^55^, ^56^ A unique case of *ZC3H7B/BCOR‐*positive HG‐ESS was identified at an early stage when an endocervical polypoid mass from the lower uterine segment was examined.^56^ Though macroscopically the tumor presented as a polypoid mass descending into the cervical canal in a myoma nascens‐like fashion, the histomorphologic and immunohistochemical profiles were suggestive of HG‐ESS.

Recently, additional variant partners for *BCOR*‐fusions, including *L3MBTI2*, *EP300*, *NUTM2G*, *RALGPS1*, *MAP7D2*, *RGAG1*, *ING3*, *NUGGC*, *KMT2D*, and *CREBBP*, were identified in a series of 40 uterine sarcomas.^57^ However, until a sufficient number of tumors showing these fusions are collected and their clinical‐pathological parameters examined in detail and reported, it is difficult to correlate meaningfully the genetic and morphologic features.

In addition to fusion genes, other types of molecular aberrations have also been found in HG‐ESS. Chiang et al.^52^ reported the first HG‐ESS with *BCOR* internal tandem duplication (ITD), the same aberration previously found in clear cell sarcoma of the kidney (CCSK)^58^, ^59^ and primitive myxoid mesenchymal tumor of infancy.^60^ This adds to the growing body of histologic, immunophenotypic, and genetic evidence unifying these tumors pathogenetically with CCSK as well as soft tissue round cell sarcomas.^47^, ^52^, ^59^ The latter tumor type also shows alternative gene fusions involving *BCOR*, such as, *BCOR/CCNB3*, *BCOR/MAML3*, and *KMT2D/BCOR*.^60^, ^61^ *BCOR* gene aberrations have further been found in a tumor of the sinonasal cavity (a sarcoma) and in pediatric glioma, resulting in *CIITA/BCOR* and *EP300/BCOR* fusion, respectively.^62^, ^63^ Truncating mutations or gene deletions occurring in *BCOR* have also been identified in acute myeloid leukemia, retinoblastoma, diffuse glioma, and medulloblastoma.^64^, ^65^, ^66^ The detection of aberrations of this gene already plays a key role in the diagnosis of some malignancies, for example, high‐grade neuroepithelial tumor of the central nervous system with *BCOR* gene alteration.^67^ *BCOR* ITD has been reported in a limited number of HG‐ESS; however, this aberration seems to characterize a younger group of patients and be associated with a slightly more favorable clinical course.^68^ The identification of the same *BCOR* genetic aberrations in so many different tumor types suggests a central pathogenetic role of this gene. In all likelihood, the type of stem cell hit by the tumorigenic event, the differentiation pattern it is already locked onto, decides the phenotypic differences observed.

The gene's orientation in the different chimeric fusions is intriguing, whether it is 3′ or 5′. Furthermore, some but not all *ZC3H7B/BCOR* positive HG‐ESS are characterized by the presence of also the reciprocal transcript^51^, ^52^, ^56^ whose role in tumorigenesis and/or progression is still unknown. Additionally, a new *BCOR*‐rearranged HG‐ESS was recently reported by Kommoss et al.^69^ in which RNAseq identified a fusion between *BCOR* and *LPP*. The genic event behind the transcript resulted in overexpression of the C‐terminal, truncated BCOR protein but without generation of the chimera, that is, no coding part of LPP was involved. The functional consequences of this aberration are unclear.

BCOR immunochemical staining has proved to be a highly sensitive marker for HG‐ESS bearing *YWHAE/NUTM2* and *YWHAE*‐rearrangements, be it with classical or unusual morphology; it was found positive in half of the tumors showing *BCOR*‐rearrangements as well as in tumors showing *BCOR* ITD.^47^, ^52^ Overexpression of *BCOR* mRNA has been described in tumors with *YWHAE/NUTM2* fusions.^48^ *BCOR* immunohistochemistry can be used diagnostically to separate all the above‐mentioned tumors (with *BCOR* genetic rearrangement) from their histological mimics.

As for the LG‐ESS, genetic aberrations found in HG‐ESS have been found also in other tumor types. Of particular interest are the data reported by Cotzia et al.^70^ who detected rearrangements of *YWHAE*, *BCOR*, and *PHF1* in a series of tumors previously classified as UUS based on morphologic and immunohistochemical features. Consequently and logically, the authors suggested that many UUS may represent misdiagnosed HG‐ESS.^70^ Stewart et al.^71^ identified a *YWHAE* deletion in a vagina wall metastasis from a monomorphic undifferentiated sarcoma, as the tumor was classified at that time. Also, two tumors with morphologic feature of LG‐ESS have had *YWHAE* rearrangements: a *YWHAE/NUTM2A* fusion was identified in a tumor confined within the endometrium,^72^ whereas deletion of a 3′ probe for *YWHAE* was shown in an LG‐ESS and in its recurrence in a case showing progression from LG‐ to HG‐ESS.^73^ Antonescu et al.^31^ identified *ZC3H7B/BCOR* in ossifying fibromyxoid tumors showing molecular overlap with ESS.

ESS showing both LG‐ and HG‐features are rare, as are tumors initially diagnosed as LG‐ but then developing metastatic HG‐ESS.^50^, ^74^, ^75^, ^76^ These tumors harbor gene fusions that are typically associated with LG‐ESS.^77^

The identification so far of four chimeric transcripts in HG‐ESS—*YWHAE/NUTM2A/B, ZC3H7B/BCOR, EPC1/SUZ12*, and *EPC1/BCOR*‐is evidence of genetic heterogeneity also within this tumor subgroup. As highlighted above, *EPC1* is involved in LG‐ESS‐related fusions. The molecular heterogeneity even within specific pathologic entities, that is, LG‐ and HG‐ESS, as well as the fact that different entities may show the same fusion, makes the diagnosis of these tumors challenging. Some tumors may exhibit morphologic aspects of a specific entity without having any known molecular signature of it, or they show sex‐cord differentiation and/or myxoid morphology reflecting phenotypic overlapping among subgroups. A tumor classification that combines both morphologic and genetic tumor features is necessary to improve diagnostic precision as well as prognostication, while simultaneously providing pathogenetic information about the neoplastic process.

## 3 PATHOGENETIC CONSEQUENCES OF ESS‐SPECIFIC GENETIC REARRANGEMENTS

In later years, much effort has gone into the identification of molecular mechanisms behind ESS‐specific genetic rearrangements with the goal of unraveling how they contribute to tumorigenesis and, eventually, how this knowledge can lead to novel therapeutic approaches. A striking general feature of the molecular genetics of ESS is that many of the genes involved in chromosomal rearrangements are associated with chromatin modification, that is, *JAZF1/SUZ12*, *JAZF1/PHF1*, *EPC1/PHF1*, *MEAF6/PHF1*, *MBTD1*/*EZHIP*, *BRD8/PHF1*, *EPC2/PHF1*, *MBTD1/PHF1*, *JAZF1/BCORL1*, *MEAF6/SUZ12*, *EPC1/SUZ12*, *EPC1/BCOR*, and *ZC3H7B/BCOR*.

LG‐ESS harbor chromosomal rearrangements of genes, such as, *SUZ12*, *PHF1*, *EZHIP*, *MBTD1*, *EPC1*, and *EPC2* (the latter is a paralog) whose protein products are associated with chromatin remodeling complexes, the NuA4 acetyltransferase complex and the Polycomb group of Protein (PcG; mainly the Polycomb Repressive Complex 2 subunits). It was recently demonstrated that the 5′ partner gene of the fusion codes for a component of the NuA4 acetyltransferase (N‐terminal on the chimeric protein) whereas the 3′ gene codes for a PcG subunit (C‐terminal on the chimeric protein).^78^, ^79^ The *JAZF1/SUZ12* chimera was first demonstrated to inhibit apoptosis and induce proliferation rates above normal in both benign and malignant uterine tumors, although only in the malignant form was suppression of the wild type/unrearranged *SUZ12* allele identified. This led to the hypothesis that genetic progression from a benign precursor to sarcoma lay behind the suppression of the unrearranged *SUZ12* allele, starting with increased cell survival but followed by accelerated cellular proliferation upon exclusion of the second allele.^80^ A similar mechanism was seen for ESS bearing *JAZF1/PHF1* fusion with simultaneous silencing of the normal *PHF1* allele.^80^ The JAZF1/SUZ12 protein has been shown to be an essential component of the Polycomb Repressive Complex 2 (PRC2), a major player in epigenetic silencing responsible for methylation of lysines 9 and 27 of histone 3 (H3K9 and H3K27). JAZF1/SUZ12 destabilizes the PRC2 components leading to a decrease of methyltransferase activity, especially on H3K27, and therefore activates chromatin and/or genes normally repressed by PRC2.^81^ Analyses of the gene expression profiles of LG‐ESS have shown overexpression of genes directly regulated by *SUZ12* and activation of genes implicated in the Wnt signaling pathway,^82^ confirming that different chromosomal rearrangements may lead to similar gene expression profiles.^36^, ^82^ It has therefore been suggested that LG‐ESS chimeric proteins disrupt the repressive function of the PRC2 complex; possibly these chimeras contribute to overexpression of Wnt ligands with subsequent activation of the Wnt signaling pathway and formation of an active β‐catenin/Lef1 transcriptional complex.^81^, ^82^ The latter would also explain why there is nuclear expression of β‐catenin in 60% of LG‐ESS.^83^, ^84^, ^85^

*BCOR* (BCL‐6 interacting corepressor, mapping on Xp11) and *BCORL1* (BCL‐6 corepressor‐like 1), like other genes rearranged in EST, are transcriptional corepressors. Specifically, *BCOR* is part of the PRC complex and promotes transcriptional repression by covalent modification of histone deacetylases and the polycomb repressive complex 1.^86^ *BCOR* has a number of functions within normal tissue and its alteration can result in developmental disorders and a variety of hematologic and solid malignancies.^48^, ^58^, ^59^, ^87^, ^88^, ^89^, ^90^

Lately, methylation profiles for different uterine tumors have been determined^91^ showing different methylation clusters correlating with established diagnostic entities. The data obtained highlighted that the LG‐ESS pattern differed from that of HG‐ESS, and that, within the latter, distinct subgrouping of *YWHAE*‐ and *BCOR*‐rearranged tumors was possible.^91^ The copy number‐profile was investigated by the same group in a series of uterine tumors that included LG‐ESS, HG‐ESS, UTROSCT, uterine leiomyomas, and uterine leiomyosarcomas.^69^ The authors identified amplification of the *MDM2* gene from chromosomal band 12q15 only in *BCOR*‐rearranged HG‐ESS.^69^ Previously, a study by Shoolmeester et al.^92^ showed *MDM2* amplification in an LG‐ESS with *JAZF1*‐rearrangement and in a UUS. Since such amplifications have not been identified in other mesenchymal uterine tumors, it is intriguing that Cotzia et al.^70^ suggested that UUS are unrecognized HG‐ESS. The discovery of *MDM2* amplification opens up for potential use of targeted therapy in a subset of HG‐ESS.^69^

Lin et al.^57^ recently investigated the genomic profile of 40 uterine sarcomas harboring *BCOR* alterations. The analyzed tumors were found to be stable at the microsatellite level; however, some of them showed homozygous deletion of *CDKN2A* which codes for an inhibitor of *CDK4* and *CDKN2B*. Furthermore, a similar copy number profile was identified for the *CDK4*, *MDM2*, and *FRS2* genes (all located at 12q14.1) in uterine sarcomas bearing *BCOR*‐fusions, but not in tumors with *BCOR* ITD. It seems that alteration of *CDK4* pathway members contributes to the pathogenesis of *BCOR*‐rearranged tumors, something that may have therapeutic implications.^57^

Histone acetyltransferases (HAT) of the MYST family are known to be involved in vital cellular processes, such as, gene transcription, detection and repair of DNA damage, and DNA replication. They carry out a significant proportion of all nuclear acetylation, and their anomalous activity, or anomalous activity of complexes associated with them (these enzymes work in multisubunit protein complexes), can lead to different anomalies from cell death to uncontrolled growth, the latter leading to cancer formation.^93^ There are different HATs in the MYST family, many of which are known to be involved in different types of cancer, for example, *MOZ* and *MORF* in acute myeloid leukemia.

Of all chimeric proteins associated with ESS, YWHAE/NUTM2A/B is the only one that does not undergo epigenetic modification. The gene for YWHAE (14‐3‐3 ε) belongs to a broad family of proteins responsible for mediating signal transduction.^40^ FAM22A/B was renamed NUTM2A/B due to its sequence homology with NUT (NUTM1), which is notable in NUT midline carcinoma.^94^

The issue whether a linear tumor progression exists among the different EST was investigated by means of array based Comparative Genomic Hybridization (aCGH).^95^ The fact that no chromosomal aberrations were common to the ESN, LG‐ESS, and UES/UUS investigated led the authors to conclude that this proposition was unlikely. However, an increasing number of aberrations were registered from ESN to UES, correlating well with histological grading and worsening clinical behavior.^95^

## Data Availability

Data sharing is not applicable to this article as no new data were created or analyzed in this study.
